# Interferometric fluorescence cross correlation spectroscopy

**DOI:** 10.1371/journal.pone.0225797

**Published:** 2019-12-18

**Authors:** Ipsita Saha, Saveez Saffarian

**Affiliations:** 1 Center for Cell and Genome Science, University of Utah, Salt Lake City, Utah, United States of America; 2 Department of Physics and Astronomy, University of Utah, Salt Lake City, Utah, United States of America; 3 Department of Biology, University of Utah, Salt Lake City, Utah, United States of America; Pennsylvania State Hershey College of Medicine, UNITED STATES

## Abstract

Measuring transport properties like diffusion and directional flow is essential for understanding dynamics within heterogeneous systems including living cells and novel materials. Fluorescent molecules traveling within these inhomogeneous environments under the forces of Brownian motion and flow exhibit fluctuations in their concentration, which are directly linked to the transport properties. We present a method utilizing single photon interference and fluorescence correlation spectroscopy (FCS) to simultaneously measure transport of fluorescent molecules within aqueous samples. Our method, within seconds, measures transport in thousands of homogenous voxels (100 nm)^3^ and under certain conditions, eliminates photo-physical artifacts associated with blinking of fluorescent molecules. A comprehensive theoretical framework is presented and validated by measuring transport of quantum dots, associated with VSV-G receptor along cellular membranes as well as within viscous gels.

## Introduction

As our understanding of cellular environments advance, the non-equilibrium, non-steady state nature of chemical reactions in biology becomes apparent [1–5]. Since these reactions are not at equilibrium, transport needs to be measured simultaneously across the sample to uncover correlations and complex relationships. Measurements of transport in microscopic systems and cells have advanced significantly using single particle tracking methods [6–10] and with the application of high resolution localization techniques, it has been possible to track well defined molecules or molecular assemblies with nanometer precision within live cells [11, 12]. Meanwhile simultaneously measuring three dimensional diffusion and flow within the whole sample without identifiable traceable objects has remained out of reach of the particle tracking methods.

Diffusion and flow within the sample can be measured by analyzing the fluctuations in fluorescence due to underlying transport properties using Fluorescence Correlation Spectroscopy (FCS) [13]. In recent years, FCS has advanced by introduction of cross correlation [14] as well as line scanning and pair correlation methods [15–17]. Unlike particle tracking which requires resolving single particles, correlation spectroscopy can distill transport properties through fluctuations in a fluorescence signal corresponding to many molecules. Variations of FCS have been used to gather basic transport properties as well as connectivity maps of various compartments within live cells as [18, 19] reviewed in [20]. The correlation spectroscopy methods are however limited by the low optical resolution along the optical axis as well as limited number of voxels analyzed during the experiments.

Interferometric fluorescence measurements were first introduced in interferometric PALM microscopy in 2009 [21]. In these microscopes the photon wave-front interferes with itself with varying phase shifts and is imaged on multiple cameras. The result of using interference has been a significant increase in effective resolution of the optical microscope which now allows a routine ~ (10*nm*)^3^ resolution for localizing single molecules within the sample [22].

Here we have merged Fluorescence Cross Correlation Spectroscopy with interferometric single photon localizations to simultaneously measure transport in a cross section of the sample in 200×200 voxels with a voxel resolution of (100 *nm*)^3^. In our current setup, we can resolve transport along plasma membrane and or within viscous gels, however, due to limitations of detector speeds, we are unable to create a transport map of the cytosol of living cells. We discuss new detector technologies which should allow cytosolic measurements.

## Results

### Experimental setup

**Fig 1:**
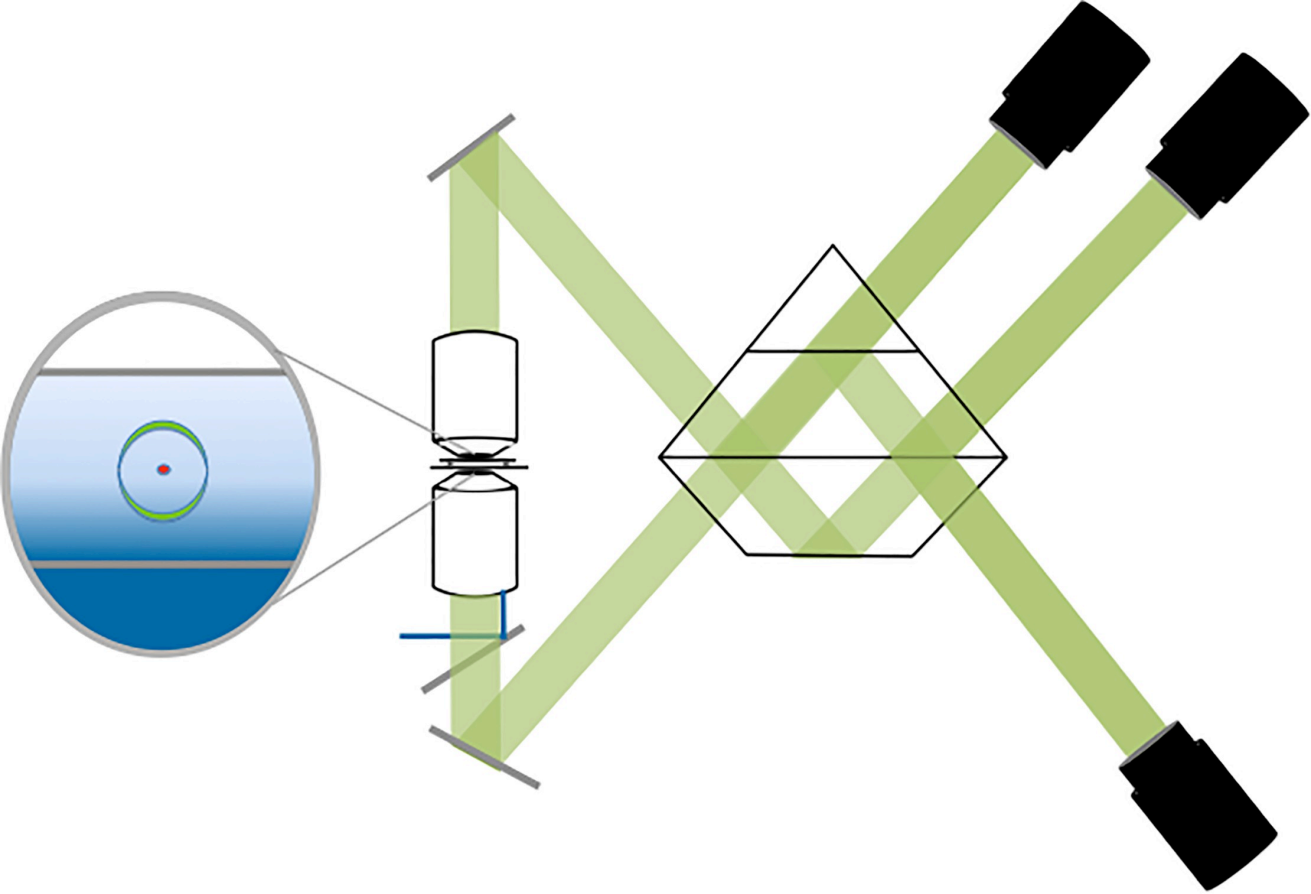
The experimental setup. The setup is composed of two Nikon 60X Apo TIRF objectives of NA 1.49 focused on the sample from top and bottom. The sample is illuminated by a 315 mW 561 nm laser (shown in blue) focused on the back aperture of the lower objective to generate TIRF illumination as shown. The custom 3-way beam splitter was adjusted so as to get the interference 0^0^, 120^0^ and 240^0^ phase shift between the cameras (Shown in black, Hamamatsu Orca Flash 4.0 sCMOS). The green lines outline the fluorescence path through the instrument and prism until it reaches the three cameras.

Our instrument is composed of two objectives focused on the sample from top and bottom as shown in the instrument diagram presented in Fig 1. In this geometry the sample is sandwiched between two coverslips and secured onto a micro positioning stage. The wave front emitted by the photon within the sample travels through two independent light paths initiated by the two objectives and is recombined in a three way prism with three phase variations of 0^0^, 120^0^ and 240^0^ each focused on an independent sCMOS camera para-focal with the sample plane.

As long as the length of the two light paths remains within the coherence length of the emitted photon ($\frac{\lambda^{2}}{n\Delta \lambda }∼5\;\mu m$ in which *λ* is the wavelength of the emitted fluorescence and *n* is the index of refraction), the photon would be detected by one of the sCMOS cameras based on the interferometric probability of its detection. The point spread function of the scope is a convolution of typical optical microscope with an interferometric effect which is best described by a sine wave as defined below and experimentally verified in S1 Fig:

$$I(x,y,z)=I_{0}exp\left(-\frac{2(x^{2}+y^{2})}{\omega^{2}})exp(-\frac{2z^{2}}{\alpha^{2}\omega^{2}})(1+\sin\left(k_{p}z+\varphi \right)\right)$$ (1)

in Eq 1, *ω* is the radial distance over which the intensity drops by 1/*e*^2^ and *αω* defines the axial distance over which the intensity drops by 1/*e*^2^ and *k*_*p*_ is the phase factor that is governed by the wavelength of excitation and numerical aperture of the objectives. The *φ* value is the interferometric phase shift of each camera and in our system it is either: 0^0^, 120^0^ and 240^0^. An experimental measurement of this point spread function is presented in S1 Fig. Based on the point spread function, when a molecule travels by 80 nm along the optical axis its fluorescence would be detected on a different camera.

### Theoretical framework of iFCCS

For molecular transport, the probability of finding a molecule at any position is governed by the Smoluchowski equation:

$$\frac{\partial p}{\partial t}=D\nabla^{2}p+v.\nabla p$$ (2)

In which *D* is the diffusion coefficient and *v* is the flux vector at any given position.

The correlation of fluorescence signal from molecules traveling as described by Smoluchowski equation are explicitly derived in the supplementary materials, briefly the correlation functions are calculated based on probability density functions convoluted with the interferometric PSF function as defined in Eq (1) and the total internal reflection excitation intensity profile Iexcitation=e−zd where *d* is the TIRF penetration depth. The generalized cross correlation function between the fluorescence detected from position (*x*_1_,*y*_1_,*φ*_1_) and (*x*_2_,*y*_2_,*φ*_2_) is calculated as:

$$G_{\left(x_{1},y_{1},\varphi_{1}\right)}^{\left(x_{2},y_{2},\varphi_{2}\right)}(\tau )=1+\frac{exp(-\frac{{\left(v_{x}\tau +\Delta_{x}\right)}^{2}}{\omega^{2}(1+\frac{\tau }{\tau_{D}})})exp(-\frac{{\left(v_{y}\tau +\Delta_{y}\right)}^{2}}{\omega^{2}(1+\frac{\tau }{\tau_{D}})})exp(-\frac{{\left(v_{z}\tau \right)}^{2}}{{\alpha^{2}\omega }^{2}(1+\frac{\tau }{{\alpha^{2}\tau }_{D}})})}{m(1+\frac{\tau }{\tau_{D}}){\left(1+\frac{\tau }{\alpha^{2}\tau_{D}}\right)}^{\frac{1}{2}}(1-exp(-\frac{\gamma }{2})\sin\left(\frac{\gamma^{\prime }}{2}\right))(1-exp(-\frac{\gamma }{2})\sin\left(\frac{\gamma^{\prime }}{2}–\varphi \right))}\times \left\{1-exp(-\frac{\gamma (1+\frac{2\tau }{\alpha^{2}\tau_{D}})}{\left(1+\frac{\tau }{\alpha^{2}\tau_{D}}\right)})\sin(\frac{\sqrt{\gamma }(v_{z}\tau )}{\left(1+\frac{\tau }{\alpha^{2}\tau_{D}}\right)}+\frac{\gamma^{\prime }}{2})+exp(-\frac{\gamma (1+\frac{2\tau }{\alpha^{2}\tau_{D}})}{\left(1+\frac{\tau }{\alpha^{2}\tau_{D}}\right)})\sin(\frac{\sqrt{\gamma }(v_{z}\tau )}{\left(1+\frac{\tau }{\alpha^{2}\tau_{D}}\right)}-\frac{\gamma^{\prime }}{2}+\varphi )-\frac{1}{2}exp(-\gamma )\cos\left(\gamma^{\prime }-\varphi \right)+\frac{1}{2}exp(-\frac{\gamma }{\left(1+\frac{\tau }{\alpha^{2}\tau_{D}}\right)})\cos\left(\frac{2\sqrt{\gamma }(v_{z}\tau )}{\left(1+\frac{\tau }{\alpha^{2}\tau_{D}}\right)}+\varphi \right)\right\}$$ (3)

In which: $\tau_{D}=\frac{\omega^{2}}{4D},\;\gamma =\frac{k_{p}^{2}\alpha^{2}\omega^{2}}{4},\;\gamma^{\prime }=\frac{k_{p}\alpha^{2}\omega^{2}}{2d}$, Δ_*x*_ = *x*_2_−*x*_1_, Δ_*y*_ = *y*_2_−*y*_1_, *φ* = *φ*_2_−*φ*_1_ and *m* is the constant depending on the concentration of molecules in the observation volume. The theoretically derived auto and cross correlation functions of a particle that moves between the cameras has been demonstrated in S2 Fig.

iFCCS works by calculating auto and cross correlation of the fluorescence signal registered on the three cameras as theoretically detailed in the supplementary materials. While in principle correlation functions can be calculated based on individual pixels, fluorescence from a single molecule is spread along an area of 3×3 pixels and therefore the fluorescence associated with each region of interest is calculated by summing the fluorescence within a mask of 3×3 pixels with the center pixel at the center of the region of interest, this is in agreement with the optimal pinhole size defined previously for FCS measurements [23]. The background is calculated based on the average fluorescence from the 200×200 pixels and subtracted from the total fluorescence intensity calculated in each region of interest as described in the supplementary section.

Experimental cross correlation functions are calculated by multiplying the signal from two ROI’s characterized by their center position (*x*,*y*) and phase *φ* of their corresponding sCMOS chip:

$$\mathrm{Correlation}\;\mathrm{function}:\;G_{\left(x_{1},y_{1},\varphi_{1}\right)}^{\left(x_{2},y_{2},\varphi_{2}\right)}(\tau )=\frac{\frac{\sum_{i=1}^{N-\tau }F_{i}{F\prime }_{i+\tau }}{N-\tau }}{\left(\frac{\sum_{i=1}^{N-\tau }F_{i}}{N-\tau })(\frac{\sum_{j=1}^{N-\tau }{F\prime }_{j+\tau }}{N-\tau }\right)}$$ (4)

where $G_{\left(x_{1},y_{1},\varphi_{1}\right)}^{\left(x_{2},y_{2},\varphi_{2}\right)}(\tau )$ is the cross correlation function, *N* is the total number of frames, *F* is the integrated fluorescence minus background from the 9 pixel ROI at (*x*_1_,*y*_1_,*φ*_1_) and *F*′ is the integrated fluorescence minus background from the 9 pixel ROI at (*x*_2_,*y*_2_,*φ*_2_). The correlation functions were calculated using a multi-tau correlation algorithm [24]. In each region, 1 auto correlation function and 8 cross correlation functions are calculated. The cross correlations are between ROIs separated along the X and Y by 2 pixels, and 2 cross correlations along the optical direction between two 120^0^ phase shifted images.

### Validation of iFCCS using Monte-Carlo simulations

**Fig 2:**
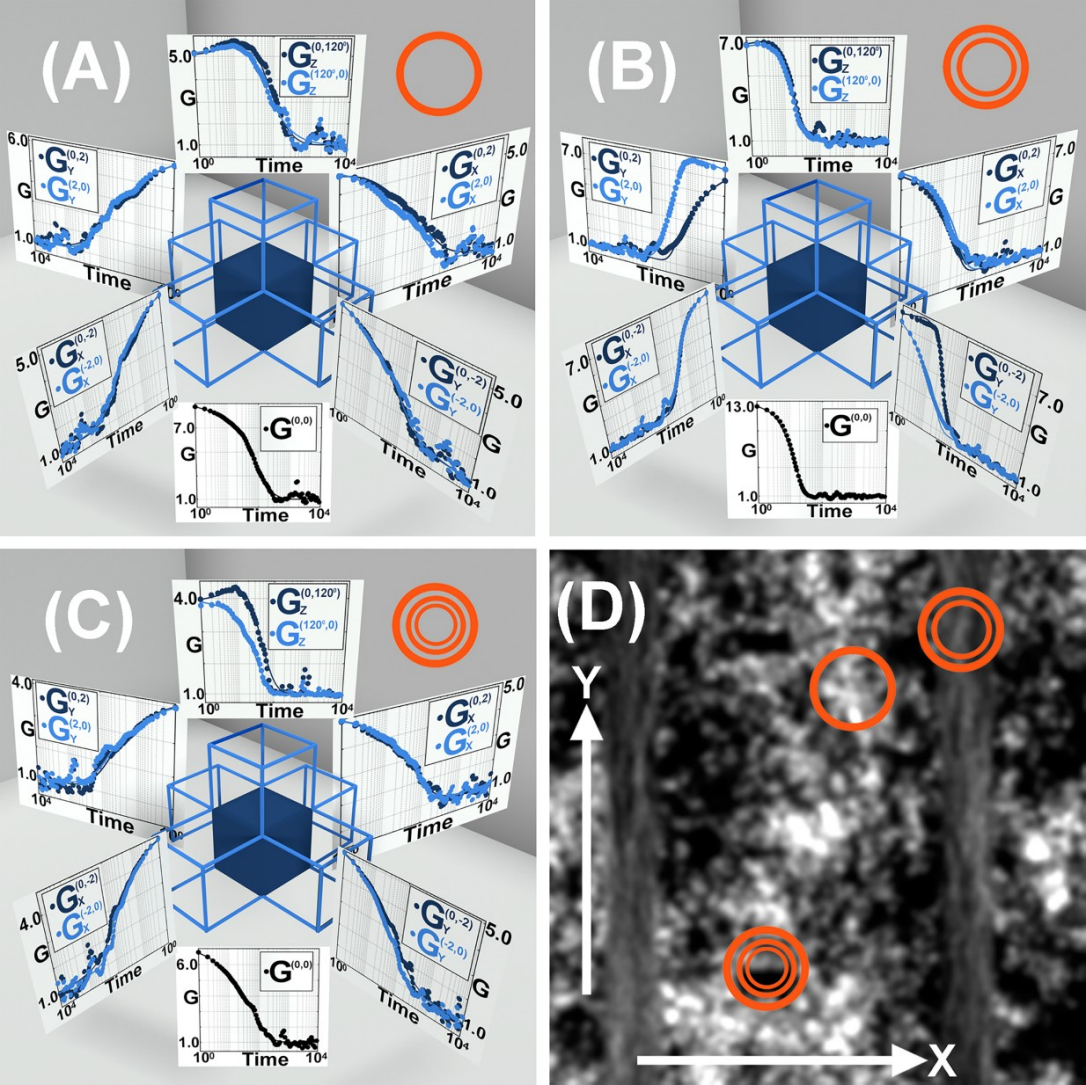
Transport measurements on particles in a box simulated by MonteCarlo dynamics. (A,B and C) show the iFCCS results from corresponding ROI highlighted by concentric circles shown in D. In each ROI, 1 auto correlation function along with 8 cross correlation functions along the three Cartesian axes is shown. For brevity, the Cross correlation functions defined in the text are simplified as $G_{\left(x_{1},0,0\right)}^{{(x}_{2},0,0)}=G_{x}^{\left(x_{1},x_{2}\right)},\;G_{\left({0,y}_{1},0\right)}^{{(0,y}_{2},0)}=G_{y}^{\left(y_{1},y_{2}\right)},$ $G_{\left({0,0,\varphi }_{1}\right)}^{{(0,0,\varphi }_{2})}=G_{z}^{\left(\varphi_{1},\varphi_{2}\right)}$ and $G_{\left(0,0,0\right)}^{\left(0,0,0\right)}=G^{\left(0,0\right)}$. (A) shows the calculated correlation functions in a region where the particles undergo pure diffusion (*D* = 10^−1^ *μm*^2^/*sec*). (B) Shows the correlation functions in a region where the particles were subjected to flow (*v* = 0.4 *μm*/*s*) along the Y axis. (C) Shows the correlation functions in a region where the particles are subjected to flow along the Z axis. (D) Shows the superimposed image from the simulated system for 20,000 frames with the regions marked which have been used for calculation in (A), (B) and (C). This overlapping image demonstrates the inhomogeneous nature of the sample. The fitted correlation curves give a diffusion coefficient of (0.89±0.048)×10^−1^ *μm*^2^/*sec* and flux of (0.36±0.028) *μm*/*sec*. For a better visualization and understanding each individual graph has been presented in the supplementary section in S4 Fig.

To validate the theoretical derivations presented above, we simulated the transport of molecules within a closed box with Monte-Carlo simulations. The details of these simulations are presented in the supplementary section. In brief, a box with dimensions of 25×25×1 *μm*^3^ was set up with reflective boundary conditions and images from the molecules moving within the box were calculated based on our microscopes PSF and camera pixel sizes (110 nm projected pixel size in sample) within the system. To visualize both diffusive transport as well as flow within the box, 4 areas within the Box were setup with distinct flow vectors. This arrangement supported steady state conditions within the box where the flow vectors canceled each other’s overall effect and the distribution of molecules as presented in the supplementary S3 Fig remained in steady state over the course of simulations. The calculated correlation functions and the simulated data are presented in Fig 2.

The amount of data generated by an iFCCS measurement is large, to streamline the presentation, we routinely show the forward and reverse cross correlation functions between a selected ROI and ROIs separated by 2 pixels along X, Y as well as forward and reverse cross correlations between phase shifted cameras from the same ROI. The nomenclature for designation of these cross correlations is defined in Eq 4. In the figure the cross correlations from the same ROI are demonstrated as a 3D plot to facilitate the communication of the data. While the 3D representation of correlation functions is useful for grasping an overall view of the transport, we present all correlation functions present in Fig 2 again in S4 Fig as independent graphs for easier analysis. The regions in which the forward and reverse correlation functions are symmetrical represent areas where the particles underwent pure diffusion while a discrepancy between forward and reverse cross correlations demonstrate directional flow of the particles. The correlation curves were fitted with the theoretical curves as derived in Eq 3 and we obtained a diffusion coefficient of (0.89±0.048)×10^−1^ *μm*^2^/*sec* and flux of (0.36±0.028) *μm*/*sec*.

### Validation of iFCCS using 3D transport of quantum dots in sucrose gels

The iFCCS method is based on interferometric localization of photons on parafocal cameras. A major prediction of this interferometric effect is the shifting of the image of a particle between two cameras as it moves approximately 80 nm along the optical axis. This effect is visualized in S2 Fig and is represented in the theoretical derivations of the cross correlation functions as shown in Eq 3. In correlation functions, this interferometric effect will result in an initial anti correlation of fluorescence signal between the cameras. Previously such anti-correlations were observed in 2D using PCF analysis. To experimentally test the presence of such anti-correlative behavior in 3D, we measured the 3D diffusion of quantum dots in a dense sucrose solution.

**Fig 3:**
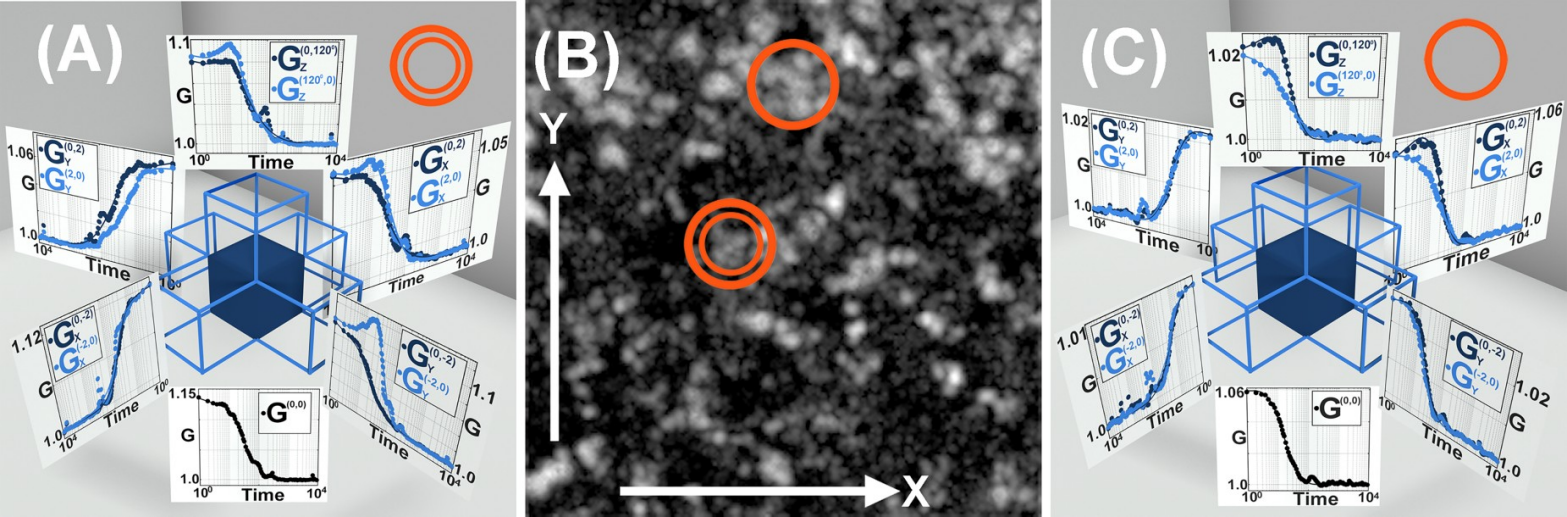
Transport measurements in a sample of quantum dots in sucrose solution. (A, B & C) show measurements of quantum dot transport within sucrose gel, (A&C) represent the correlation functions from two different regions of the sample. The cross correlation functions are defined similarly to Fig 2. (B) is the superimposed image from the experimental dataset for 20,000 frames with regions marked which have been used for calculation in (A) and (C). The correlation functions have been fitted to obtain diffusion coefficient of (2.68±0.28)×10^−1^*μm*^2^/*sec* and regions of flow of 0.21−0.55 *μm*/*sec*. For a better visualization and understanding each individual graphs has been presented in the supplementary section in S5 Fig.

Fig 3 shows correlation function analysis for 605 Quantum dots in a solution of 70% sucrose. The presentation is similar to Fig 2 as explained in the section above.

The high viscosity of the sucrose created patches of inhomogeneity within the sample. While theoretically the sucrose solution should be homogeneous, we could detect directional flow within the ROIs as characterized by the asymmetry in the measured cross correlation curves shown in the figure. These flow vectors are likely due to temperature gradients within the sample. The 15−20 *nm* diameter quantum dots in 70% sucrose should theoretically yield a diffusion coefficient of ~2×10^−1^ *μm*^2^/*sec* at 33^0^*C* and we report to have obtained a diffusion coefficient of (2.68±0.28)×10^−1^ *μm*^2^/*sec*.

### Validation of iFCCS measurements on cellular membranes

The inhomogeneous behavior of quantum dots within the sucrose gel has been slightly surprising and therefore we set out to validate the iFCCS in a system where we expect fully 2D diffusion behavior. When cells are plated on glass coverslips, their Plasma membrane near glass coverslips presents such a 2D environment. To test the iFCCS in a 2D system we performed iFCCS on VSV glycoproteins on cellular membranes. Transport of VSVG on the plasma membrane has been well characterized previously using both FCS and photo-bleaching experiments so this system posed a good test for iFCCS [25].

**Fig 4:**
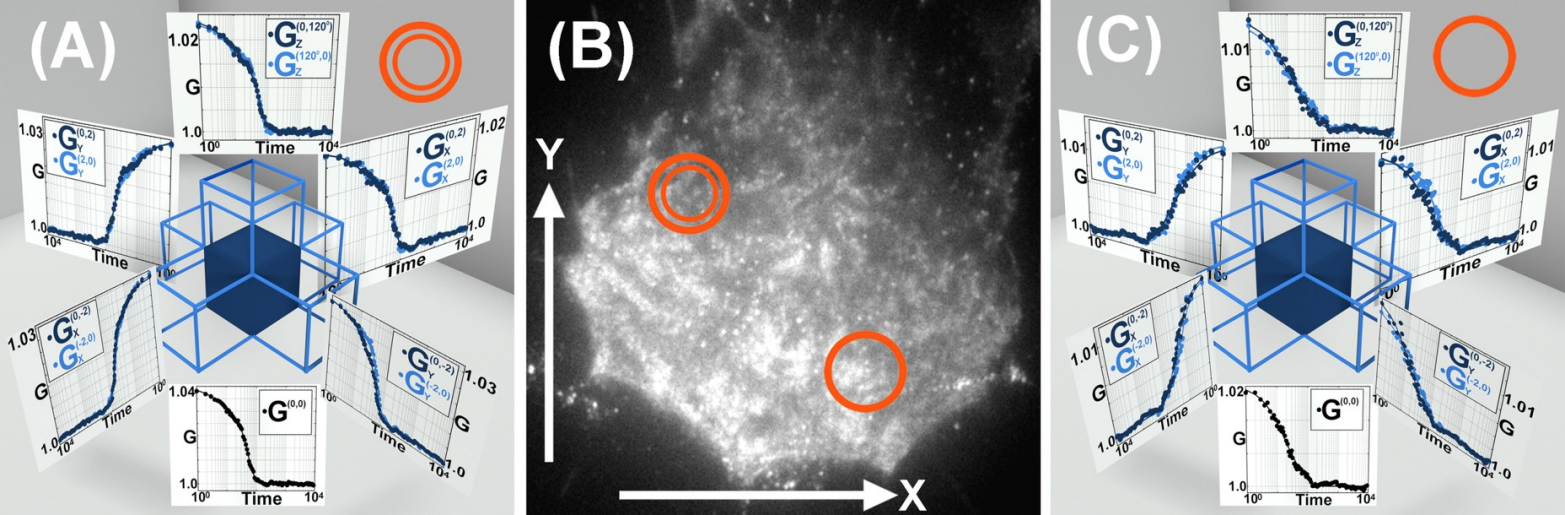
VSV-G diffusion on the membrane of HeLa cells. (A, B & C) represent measurements of transport of quantum dots associated with VSVG on the plasma membrane of HeLa cells. (A) and (C) represent the correlation functions from two different regions of the cell membrane. (B) is the image of the cell in which the correlation functions has been measured. The correlation functions report a diffusion coefficient of (1.28±1.00)×10^−2^ *μm*^2^/*sec*. The correlation function calculation and representation is same as Fig 2. For a better visualization and understanding each individual graphs has been presented in the supplementary section in S6 Fig.

Biotinylated VSV-G antibody bound to streptavidin conjugate 605 quantum dots was added to these VSV-G transfected cells. Thus the dynamics of the quantum dots reveals the diffusion of the VSV-G on the cellular membrane. Data acquired from these experiments is presented in Fig 4.

As previously explained, the experimental correlation functions from selected ROIs are shown in a similar fashion to the data presented in Figs 2 and 3 in which the relationship from each selected ROI with its neighbors can be easily visualized in the 3D correlation function plots. The correlation functions in Fig 4 reveal the 2D diffusion on the membrane as the correlation functions between two cameras show no initial anti-correlation behavior and their symmetrical nature demonstrates absence of flow on the membrane of the cells. We report to have obtained a diffusion coefficient of (1.28±1.00)×10^−2^ *μm*^2^/*sec*, which is in agreement with the previously reported value of 0.8×10^−2^ *μm*^2^/*sec*. [25]

## Discussion

Single particle methods have come a long way to measure transport across the full sample. The best such examples can be found in tracking of focal adhesions and actin assemblies during cell migration [9, 26] on a time scale of minutes. While transport measurements through particle tracking have advanced greatly, they still require the presence of identifiable particles. Correlation spectroscopy methods however by default do not require an identifiable particle and work by correlating single photons from passing molecules [2, 24, 27–29].

The iFCCS method as presented in this manuscript has the advantage of measuring transport and fluorescence cross correlation functions across a sample with homogeneous voxels. This is unique since most previous FCS based methods were limited by the lower resolution along the optical axis resulting in asymmetric aspect ratio of the observation volumes. The interferometry within iPALM is due to the interference within the fluorescence emission path, previously, standing wave two photon fluorescence correlation spectroscopy had utilized interference within the excitation path [30]. The present method of iFCCS utilizes a single TIRF excitation path. It is plausible that in future, combination of the two techniques, utilizing interference effects both in excitation and emission can result in better illumination within thicker samples not accessible through TIRF microscopy.

Our experimental validations are currently limited by the speed of the sCMOS cameras to 1 mSec per frame which limits the detection of diffusion to processes with diffusion coefficients slower than ~3×10^−1^ *μm*^2^/*sec*. This limitation however can be overcome with faster detectors, one such possibility would be SPAD detectors recently developed for other high resolution applications [31] as well as SPAD detectors specialized for multi-tau correlation spectroscopy [32]. SPAD detectors could potentially deliver frame rates on the order of microseconds and therefore allow simultaneous measurements of diffusion and flow even within the cytosol of cells. Presently the speed and noise levels of sCMOS does not allow iFCCS to be applied to organic dyes or other fluorescent proteins freely diffusing in solution due to both low frame rate as well as sensitivity.

It is informative to compare the iFCCS with PCF where cross correlations across an image were performed. One interesting feature previously described in PCF [19] is the prediction of an anti-correlation in various cross correlation situations. Aside from detecting this anti-correlation in 2D similar to OCF, we also observe this anti-correlation when the molecule travels along the optical axis resulting in a detectable anti-correlation as shown in S2 Fig.

The interferometric fluorescence cross correlation method can also be used to distinguish between true transport and chemical reactions that can affect the fluorescence emission. As an example, the quantum dots used in our studies have been shown to have blinking characteristics which can complicate the interpretation of the auto correlation functions [33]. The cross correlation curves between the detectors, which are phase shifted by 03 *φ*>0 can distinguish between fluctuations arising due to actual physical movement of the molecule and other photo-physical activity of a fluorescent proteins. This is because when the molecule blinks or goes through any other photo-physical activity it affects both the detectors simultaneously. The characteristic anti-correlation between phase shifted cameras is a signature of molecular motion along the z-axis and therefore anti-correlation as detected in Fig 2 for quantum dots diffusing in 3D can only be detected during physical movement and not any other photophysical effect. This advantage is however limited by the detector speed and is only effective when a significant anti-correlation signal is present between the detectors. If the detector speed is slow compared to the travel time between detectors, the molecule would contribute equally to both detectors and even though the molecule moves, the anti-correlation signal would not be detected.

Another distinct feature of this kind of correlation function is its asymmetry when flux is present in the system. When the system has pure diffusion the cross correlation functions are symmetric $G_{\left(x_{1},y_{1},\varphi_{1}\right)}^{\left(x_{2},y_{2},\varphi_{2}\right)}=G_{\left(x_{2},y_{2},\varphi_{2}\right)}^{\left(x_{1},y_{1},\varphi_{1}\right)}$ and when there is flux the cross correlations are asymmetric $G_{\left(x_{1},y_{1},\varphi_{1}\right)}^{\left(x_{2},y_{2},\varphi_{2}\right)}\neq G_{\left(x_{2},y_{2},\varphi_{2}\right)}^{\left(x_{1},y_{1},\varphi_{1}\right)}$. That is another advantage for determining the direction of flow of molecules in a particular observation volume.

The present form of iFCCS can be helpful in studying the dynamics involved in various biological systems both *in vitro* and *in vivo*. With proper detector speeds, a transport map of a desired molecule can be resolved within the cytosol. These observations could reveal the inhomogeneity of the various networks within cells and the iFCCS cross correlations can in principle inform about type of flow vectors and aggregation state of molecules with high resolution.

It is worth mentioning that Particle Image Velocimetry (PIV), originally a fluid mechanics discipline, operates on similar principles in order to extract the large-scale velocity field [34–36]. In fact, PIV makes use of cross-correlations among segments of successive image frames, to reconstruct macroscopic flow fields. More recently an Optimal Transport formulation of PIV (OT-PIV) has been introduced which can provide an alternative to the cross-correlation technique [37].

## Materials and methods

### Simulations

The conditions for the simulated system were kept close to the real experimental conditions. 250 particles were randomly distributed in a cube, which had a volume of 25×25×1 *μm*^3^, with reflecting boundary conditions. The pixel size in the simulation was chosen to be 100*nm*. The initial position of the particles was generated from rand function of MATLAB. Spatial inhomogeneity was created where the particles were undergoing pure diffusional motion in some region while there were regions where the particles were subjected to directional flow as shown in S3 Fig. Areas of inhomogeneity were created in a way such that steady state condition was maintained in the system. A normalized random number generator (normrnd) determined the step sizes of each particle, the mean of which depended on our flux vector (0.4 *μm*/*sec*) and the standard deviation were determined by the diffusion coefficient (10^−1^ *μm*^2^/*sec*). For the PSF, typical *ω* was chosen to be 264.5 *nm* and *α* was 4. The particles were excited by a 561 *nm* laser with a field depth of 300 *nm* and TIRF imaging conditions were maintained. Two detectors 120^0^ phase shifted detected signals from these particles in the mentioned conditions for 20,000 frames with 1 *mSec* of exposure. A poissonian random number determined the signals in the 200×200 pixel area with mean given by the fluorescence function as described in Eq 1. The simulation code was written in MATLAB and run on the compute nodes with two Intel Xeon Gold 6130 CPUs, 32 CPU cores and 96 GB of RAM per node.

### Experimental setup

Our instrument is a prototype setup from Thermo Fisher Scientific as schematically described in Fig 1. It is composed of two Nikon 60X Apo TIRF objectives of NA 1.49 focused on the sample from top and bottom. In this geometry the sample is sandwiched between two coverslips and secured on to a micro positioning stage and was illuminated by a 315 *mW* 561 *nm* laser. The 100 *nm* gold beads on the Hestzig slides were used to focus and calibrate the whole system. The custom 3-way beam splitter was adjusted so as to get the interference and 120^0^ phase shift between the cameras (Hamamatsu Orca Flash 4.0 sCMOS) were obtained as seen in the calibration curves in S1 Fig.

### Experiments

To experimentally validate our method, a sample was prepared by adding Quantum dots (Quantum dots 605, Thermo Fisher Scientific) in 70% sucrose solution. A 25 *mm* 1.5 Hestzig coverslip and an 18 *mm* 1.5 coverslip were used to sandwich the sample. The circular coverslips were thoroughly washed in 1 M sodium hydroxide solution followed by mQ water and then blow dried with nitrogen. 70% sucrose solution was prepared by dissolving the sucrose in 10 *mg*/*ml* casein solution in PBS. The sucrose solution was prepared by heating it in a water bath to 80^0^*C*. Care was taken not to insert any air bubble or to crystallize the sucrose. 605 Quantum dots, by Thermo Fisher Scientific, were added to it when the sucrose completely dissolved and produced a clear viscous solution. The Quantum dots were heated with sucrose so the sample mixes well. After proper mixing, the sample was sandwiched between the coverslips and sealed with glue and allowed to cool down to room temperature. The casein prevented the quantum dots from sticking to the coverslips. 20,000 frames were acquired on three cameras with 1 *mSec* exposure and experimental cross correlation functions identical to the arrangement in Fig 1 were calculated within the corresponding images. The superimposed image of the 20,000 frames is shown as well as correlation functions calculated within two selected ROIs as shown in Fig 3.

We also measured pure 2*D* diffusion of VSV glycoproteins on cellular membranes. The coverslips were thoroughly washed in 1 M sodium hydroxide solution followed by mQ water and then blow dried with nitrogen and plasma cleaned. After cleaning, the coverslips were kept under UV irradiation in the biosafety cabinet for 2 hours before cells were plated. HeLa cells were plated on 25 *mm* #1.5 Hestzig coverslip. The cells were transfected with VSV-G GFP, 12 hours prior to experiment. 2 *μl* of biotinylated VSV-G antibody, by abcam, and 2 *μl* of streptavidin conjugate 605 Quantum dots, by Thermo Fisher Scientific, were mixed in 20 *μl* of *CO*_2_ independent media and incubated for 5 *min*. Then this mixture was diluted in 180 *μl* of the media and added to the cells and incubated for 10 *min*. The cells were then washed with the media and sandwiched with 25 *mm* regular coverslips and vacuum grease. 20,000 frames were acquired on three cameras with 2 *mSec* exposure and cross correlation functions were calculated as shown in Fig 4. These correlation functions reveal the 2D diffusion on the membrane and their symmetrical nature demonstrates absence of flow on the membrane of the cells. We report to have a diffusion coefficient of (1.28±1.00)×10^−2^ *μm*^2^/*sec*.

## Supporting information

S1 Fig Calibration curve showing the interference effect and phase difference between the cameras. Calibration was performed by moving the sample in between the two objectives, in steps of 8*nm* for 101 planes, along the axial direction. At each plane the 3 cameras collected fluorescence signal from a fiducial.
(TIF)

S2 Fig Demonstrates the movement of the molecule between the cameras and their associated correlation functions. (TIF)

S3 Fig Represents the simulated system where the regions where the particles were subjected to flow are marked with arrows. The flow regions along the axial plane have a volume of 2X25X0.3 *μm*^3^ and the ones along the optical axis have a volume of 2X2X1 *μm*^3^.
(TIF)

S4 Fig Individual graphs for Fig 2. (TIF)

S5 Fig Individual graphs for Fig 3. (TIF)

S6 Fig Individual graphs for Fig 4. (TIF)

S1 File This contains all the supporting information. (DOCX)
