# Supplementary figures and images for: Interferometric fluorescence cross correlation spectroscopy
Source: PLoS One. 2019 Dec 18;14(12):e0225797. doi: 10.1371/journal.pone.0225797 (PMC6919592; doi:10.1371/journal.pone.0225797)

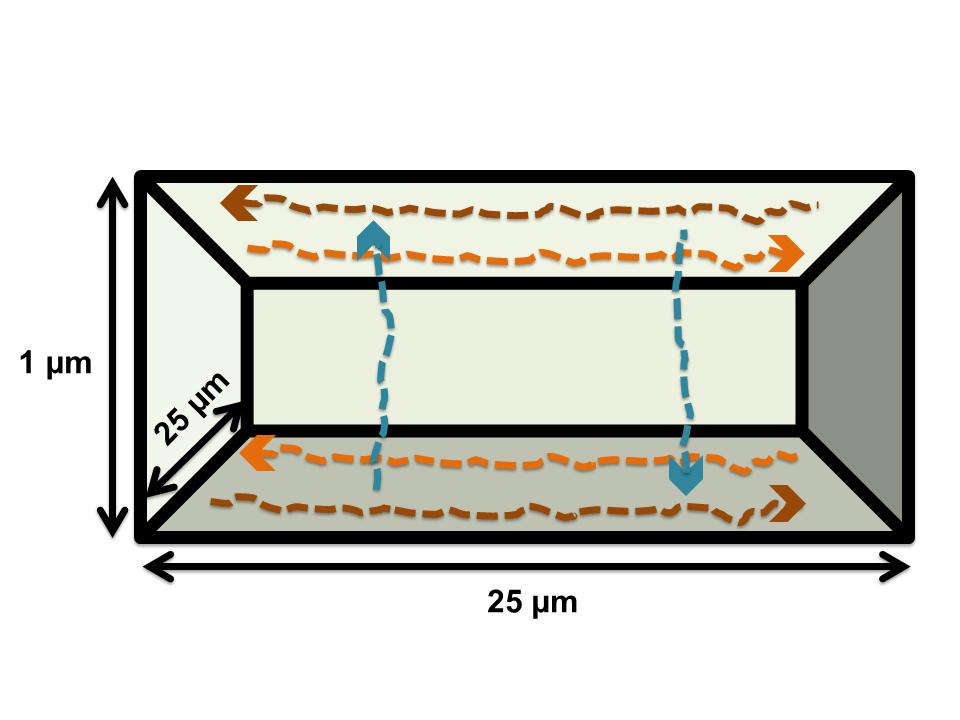

Supplement: S3 Fig — The flow regions along the axial plane have a volume of 2X25X0.3 μm3 and the ones along the optical axis have a volume of 2X2X1 μm3. (TIF) [file pone.0225797.s003.tif]

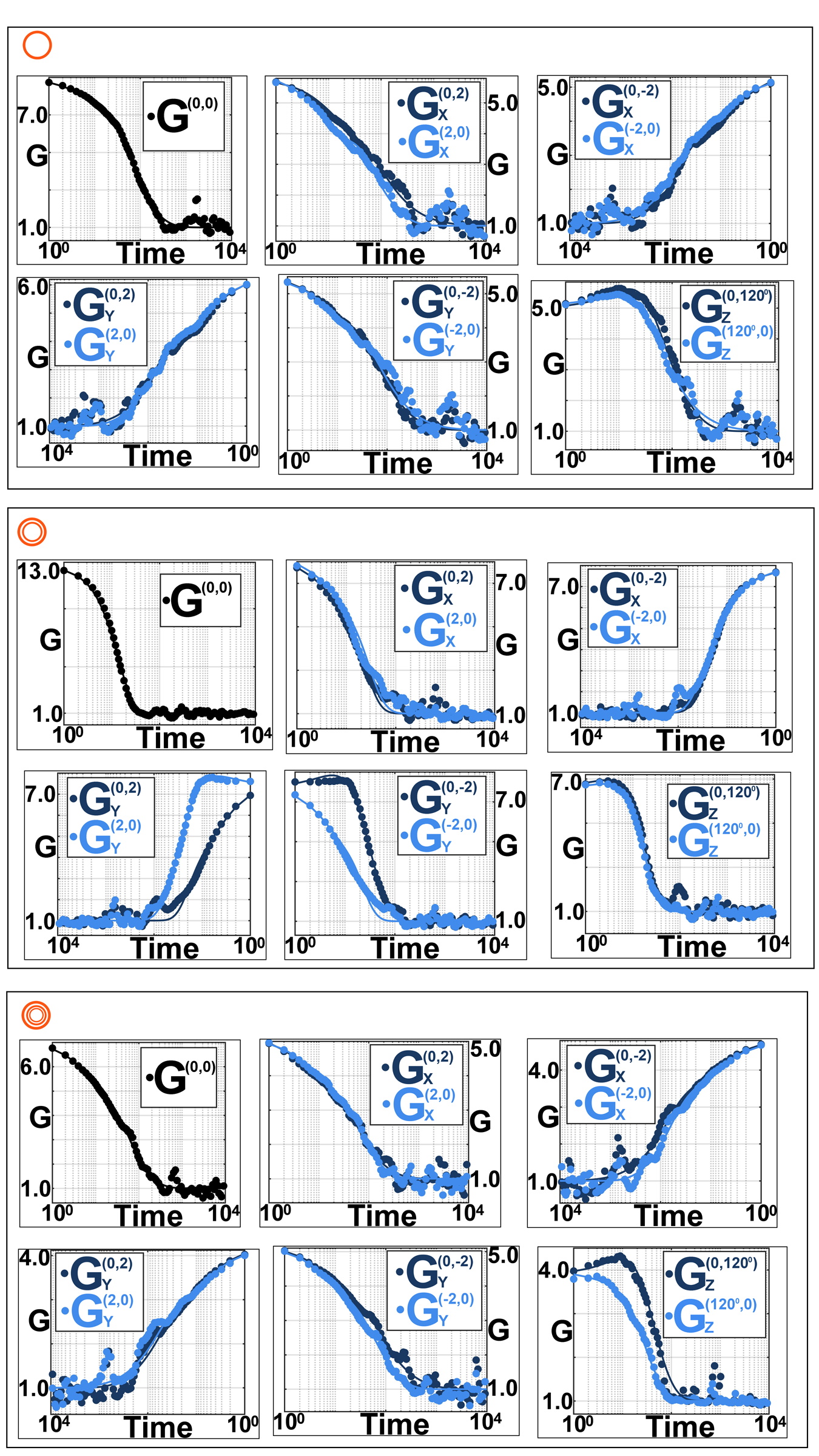

Supplement: S4 Fig — (TIF) [file pone.0225797.s004.tif]

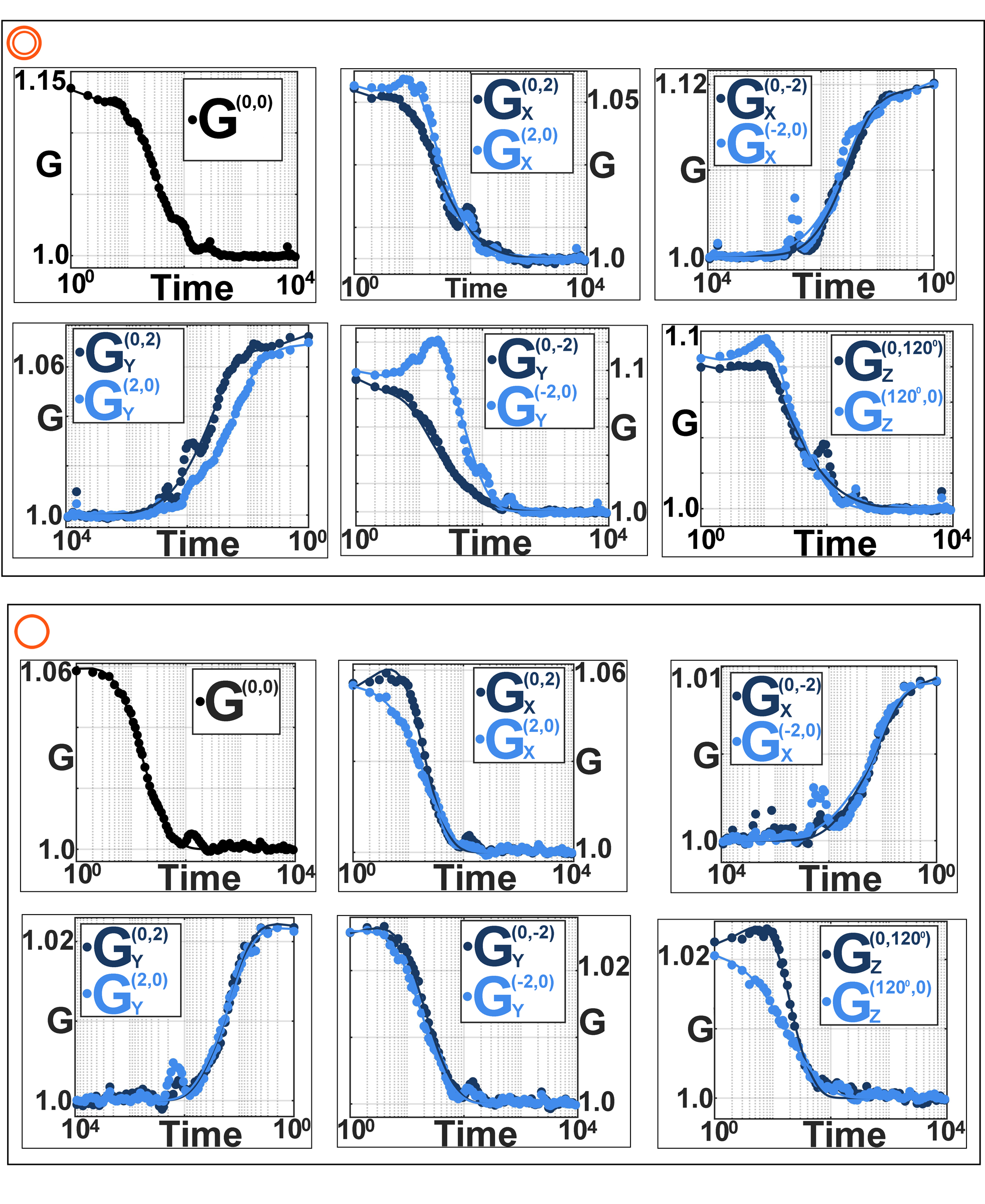

Supplement: S5 Fig — (TIF) [file pone.0225797.s005.tif]

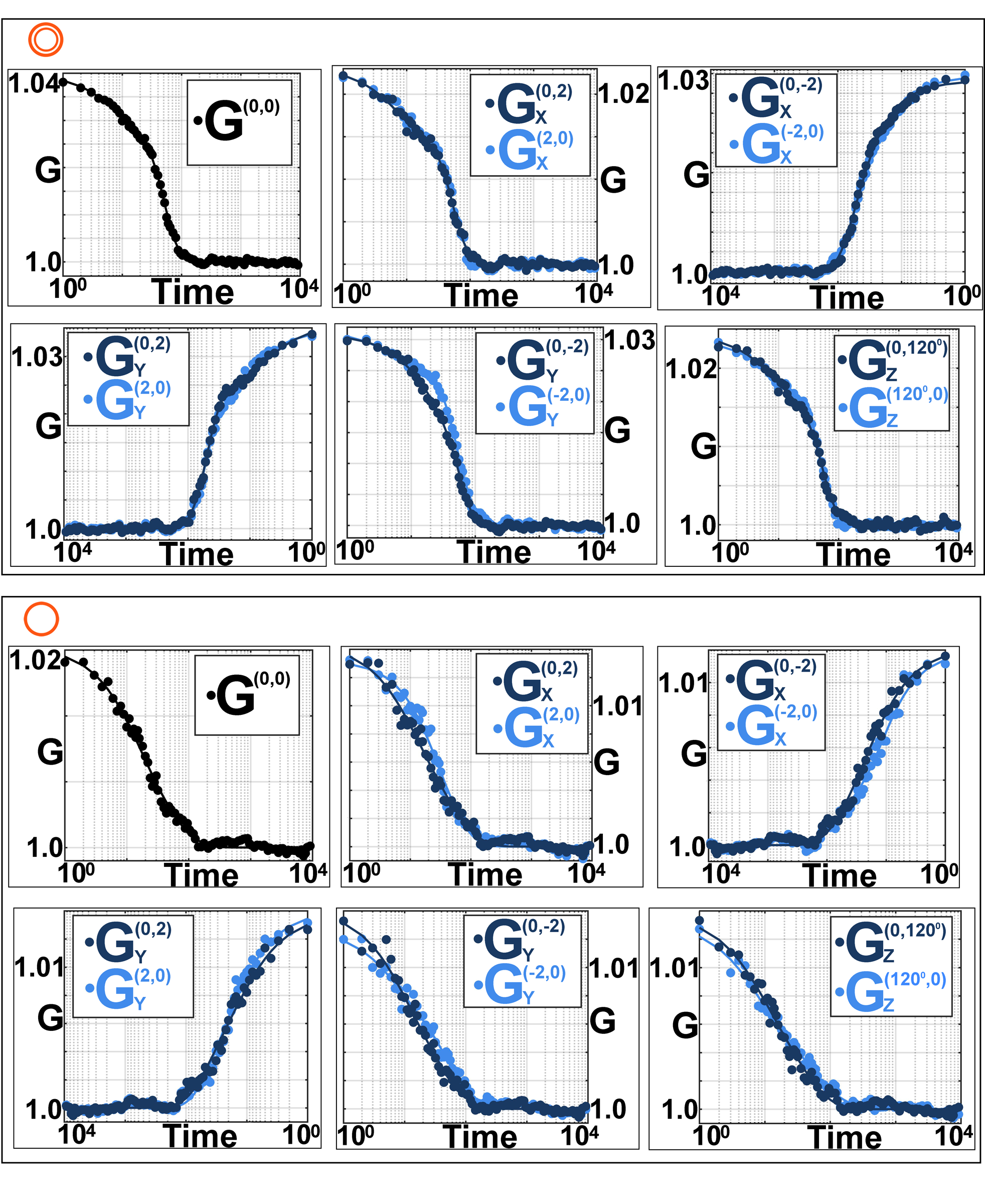

Supplement: S6 Fig — (TIF) [file pone.0225797.s006.tif]
