# Supplementary material for: Interferometric fluorescence cross correlation spectroscopy
Source: PLoS One. 2019 Dec 18;14(12):e0225797. doi: 10.1371/journal.pone.0225797 (PMC6919592; doi:10.1371/journal.pone.0225797)
Supplement: S1 File — (DOCX) [file pone.0225797.s007.docx]

**S1 text: Sample Prep**

**Quantum dots in sucrose**

A $25 mm$ $1.5$ Hestzig coverslip and an $18 mm$ $1.5$ coverslip were used to sandwich the sample. The circular coverslips were thoroughly washed in $1 M$ sodium hydroxide solution followed by mQ water and then blow dried with nitrogen. $70\%$ sucrose solution was prepared by dissolving the sucrose in $10 mg/ml$ casein solution in PBS. The sucrose solution was prepared by heating it in a water bath to ${80}^{0}C$. Care was taken not to insert any air bubble. $605$ Quantum dots, by Thermo Fisher Scientific, were added to it when the sucrose completely dissolved and produced a clear viscous solution. The Quantum dots were heated with sucrose so the sample mixes well. After proper mixing, the sample was sandwiched between the coverslips and sealed with glue and allowed to cool down to room temperature. The casein prevented the quantum dots from sticking to the coverslips.

**VSV-G HeLa cells**

The coverslips were thoroughly washed in $1 M$ sodium hydroxide solution followed by mQ water and then blow dried with nitrogen and plasma cleaned. After cleaning, the coverslips were kept under UV irradiation in the biosafety cabinet for 2 hours before cells were plated. HeLa cells were plated on $25 mm$ $\#1.5$ Hestzig coverslip. The cells were transfected with VSV-G GFP, $12$ hours prior to experiment. $2 \mu l$ of biotinylated VSV-G antibody, by abcam, and $2 \mu l$ of streptavidin conjugate $605$ Quantum dots, by Thermo Fisher Scientific, were mixed in $20 \mu l$ of $CO_{2}$ independent media and incubated for $5 min$. Then this mixture was diluted in $180 \mu l$ of the media and added to the cells and incubated for $10 min$. The cells were then washed with the media and then sandwiched with $25 mm$ regular coverslips and vacuum grease.

**S2 text: Instrument specification and experimental details**

The instrument is a prototype setup from Thermo Fisher Scientific as schematically described in Fig. S1. The sample was placed between two Nikon 60X Apo TIRF objectives of NA 1.49 and was illuminated by a $315 mW$ $561 nm$ laser. The $100 nm$ gold beads on the Hestzig slides were used to focus and calibrate the whole system. The custom 3-way beam splitter was adjusted so as to get the interference and${120}^{0}$ phase shift between the cameras (Hamamatsu Orca Flash 4.0 sCMOS) were obtained as seen in the calibration curves in Fig. S2. Data was collected on a $200\times200$ pixel area with $1 msec$ of exposure for $20,000$ frames.

**S3 text: Simulation details**

The conditions for the simulated system were kept close to the real experiments. 250 particles were taken in a cube, which had a volume of $25\times25\times1 \mu m^{3}$. The pixel size in the simulation was chosen to be $100nm$. The initial position of the particles was generated from rand function of MATLAB. The system had reflecting boundary condition. Spatial inhomogeneity was created where the particles were undergoing pure diffusional motion in some region while there were regions where the particles were subjected to directional flow as shown in figure S4. Areas of inhomogeneity were created in a way such that steady state condition was maintained in the system. A normalized random number generator (normrnd) determined the step sizes of each particle, the mean of which depended on our flux vector $(0.4 \mu m/sec)$ and the standard deviation were determined by the diffusion coefficient $({10}^{-9}cm^{2}/\sec)$. For the PSF, typical $\omega$ was chosen to be $264.5 nm$ and $\alpha$ was $4$. The particles were excited by a $561 nm$ laser with a field depth of $300 nm$ and TIRF imaging conditions were maintained. Two detectors ${120}^{0}$ phase shifted detected signals from these particles in the mentioned conditions for $20,000$frames with $1 msec$ of exposure. A poissonian random number determined the signals in the $200\times200$ pixel area with mean given by the PSF function as described in eq 1. The simulation code was written in MATLAB and run on the compute nodes with two Intel Xeon Gold 6130 CPUs, 32 CPU cores and 96 GB of RAM per node.

**S4 text: Mathematical details of the theoretical development**

The point spread function of the scope is a convolution of typical optical microscope with an interferometric effect which is best described by a sine wave as defined below and experimentally verified in Figure S2:

$I\left( x,y,z \right)=I_{0}exp \left( -\frac{2\left( x^{2}+y^{2} \right)}{\omega^{2}} \right)exp\left( -\frac{2z^{2}}{\alpha^{2}\omega^{2}} \right)\left( 1+\sin\left( k_{p}z+\varphi\right) \right)$ (1)

In which $\omega$ is the radial distance over which the intensity drops by $1/e^{2}$ and $\alpha\omega$ defines the axial distance over which the intensity drops by $1/e^{2}$ and $k_{p}$is the phase factor that is governed by the wavelength of excitation and numerical aperture of the objectives. The $\varphi$ value is the interferometric phase shift of each camera and in our system it is either: $0^{0}$, ${120}^{0}$ and ${240}^{0}$.

The probability of finding a molecule at any position is governed by the Smoluchowski equation:

$\frac{\partial p}{\partial t}=D\nabla^{2}p+v.\nabla p$ (2)

In which $D$ is the diffusion coefficient and $v$ is the flux vector of the molecule.

Fluorescence Correlation functions are calculated based on probability density functions convoluted with the interferometric PSF function as defined in equation (1) and the total internal reflection excitation intensity profile $I_{excitation}=e^{\frac{-z}{d}}$ where $d$ is the TIRF penetration depth. Following the derivations as explicitly demonstrated in the supplementary, the generalized cross correlation function between the fluorescence detected from position $(x_{1},y_{1},\varphi_{1})$ and $(x_{2},y_{2},\varphi_{2})$ is calculated as:

$G_{\left( x_{1},y_{1},\varphi_{1} \right)}^{\left( x_{2},y_{2},\varphi_{2} \right)}(\tau)=1+\frac{exp\left( -\frac{\left( v_{x}\tau+\Delta_{x} \right)^{2}}{\omega^{2}\left( 1+\frac{\tau}{\tau_{D}} \right)} \right)exp\left( -\frac{\left( v_{y}\tau+\Delta_{y} \right)^{2}}{\omega^{2}\left( 1+\frac{\tau}{\tau_{D}} \right)} \right)exp\left( -\frac{\left( v_{z}\tau\right)^{2}}{{\alpha^{2}\omega}^{2}\left( 1+\frac{\tau}{{\alpha^{2}\tau}_{D}} \right)} \right)}{m\left( 1+\frac{\tau}{\tau_{D}} \right)\left( 1+\frac{\tau}{\alpha^{2}\tau_{D}} \right)^{\frac{1}{2}}\left( 1-exp\left( -\frac{\gamma}{2} \right)\sin\left( \frac{\gamma^{'}}{2} \right) \right)\left( 1-exp\left( -\frac{\gamma}{2} \right)\sin\left( \frac{\gamma^{'}}{2} - \varphi\right) \right)}\times$ (3)

$$\left\{ 1-exp\left( -\frac{\gamma\left( 1+\frac{2\tau}{\alpha^{2}\tau_{D}} \right)}{\left( 1+\frac{\tau}{\alpha^{2}\tau_{D}} \right)} \right)\sin\left( \frac{\sqrt{\gamma}\left( v_{z}\tau\right)}{\left( 1+\frac{\tau}{\alpha^{2}\tau_{D}} \right)}+\frac{\gamma^{'}}{2} \right)+exp\left( -\frac{\gamma\left( 1+\frac{2\tau}{\alpha^{2}\tau_{D}} \right)}{\left( 1+\frac{\tau}{\alpha^{2}\tau_{D}} \right)} \right)\sin\left( \frac{\sqrt{\gamma}\left( v_{z}\tau\right)}{\left( 1+\frac{\tau}{\alpha^{2}\tau_{D}} \right)}-\frac{\gamma^{'}}{2}+\varphi\right)-\frac{1}{2}exp\left( -\gamma\right)\cos\left( \gamma^{'}-\varphi\right) +\frac{1}{2}exp\left( -\frac{\gamma}{\left( 1+\frac{\tau}{\alpha^{2}\tau_{D}} \right)} \right)\cos\left( \frac{2\sqrt{\gamma}\left( v_{z}\tau\right)}{\left( 1+\frac{\tau}{\alpha^{2}\tau_{D}} \right)}+\varphi\right) \right\}$$

In which: $\tau_{D}=\frac{\omega^{2}}{4D}$, $\gamma=\frac{k_{p}^{2}\alpha^{2}\omega^{2}}{4}$, $\gamma^{'}=\frac{k_{p}\alpha^{2}\omega^{2}}{2d}$, $\Delta_{x}=x_{2}-x_{1}$,$\Delta_{y}=y_{2}-y_{1}$, $\varphi=\varphi_{2}-\varphi_{1}$ and $m$ is the constant depending on the concentration of molecules in the observation volume.

For a system of volume $V$, the probability of the molecule being at $\left( x^{'},y^{'},z^{'} \right)$ at $t=0$,

$P\left( x^{'},y^{'},z^{'} \right)=\frac{1}{V}$

The fluorescence signal received by a detector (detector 1) from this molecule

$$I\left( x^{'},y^{'},z^{'} \right)=I_{0}exp \left( -\frac{2\left( x^{'2}+y^{'2} \right)}{\omega^{2}} \right)exp\left( -\frac{z^{'}}{d} \right)exp\left( -\frac{2z^{'2}}{\alpha^{2}\omega^{2}} \right)\left( 1+\sin\left( k_{p}z^{'} \right) \right)$$

Since the molecule is has both diffusion as well as a directional flow, the probability of finding the molecule at $\left( x, y, z \right)$, at $t=\tau$ can be found by solving Smoluchowski equation,

$\frac{\partial p}{\partial t}=D\nabla^{2}p+v.\nabla p$

where $D$is diffusion coefficient and $v$ is flux vector. Thus, the probability distribution function is $P\left( x,y,z \right)=\left( \frac{\pi}{2D\tau} \right)^{3/2}exp\left( -\frac{\left( v_{x}\tau+x-x^{'} \right)^{2}+\left( v_{y}\tau+y-y^{'} \right)^{2}+\left( v_{z}\tau+z-z^{'} \right)^{2}}{4D\tau} \right)$

Due to the flux the fluorescence signal received at $\left( x+\Delta_{x}, y+\Delta_{y}, z \right)$ from the same molecule by a detector which is phase shifted by $\varphi$ from detector 1 is:

$$I\left( x,y,z \right)=I_{0}exp\left( -\frac{2({(x+\Delta_{x})}^{2}+({y+\Delta_{y})}^{2})}{\omega^{2}} \right)exp\left( -\frac{z}{d} \right)exp\left( -\frac{2z^{2}}{\alpha^{2}\omega^{2}} \right)\left( 1+sin(k_{p}z+\phi) \right)$$

- The generalized correlation function can be calculated as:
- $G\left( \tau\right)=1+\frac{\left\langle p\left( t \right)p^{'}(t+\tau) \right\rangle}{M\left\langle p(t) \right\rangle\left\langle p^{'}(t) \right\rangle}$
- $G(\tau)=1+\frac{\left( \int_{-\infty}^{\infty} P\left( x^{'},y^{'}z^{'} \right)qI\left( x^{'},y^{'},z^{'} \right)P\left( x,y,z \right)qI\left( x,y,z \right){dx}^{'}{dy}^{'}{dz}^{'}dxdydz \right)}{M\left( \int_{-\infty}^{\infty} P\left( x^{'},y^{'}z^{'} \right)qI\left( x^{'},y^{'},z^{'} \right){dx}^{'}{dy}^{'}{dz}^{'} \right)\left( \int_{-\infty}^{\infty} P\left( x,y,z \right)qI\left( x,y,z \right)dxdydz \right)}$
- $G\left( \tau\right)=1+\frac{exp\left( -\frac{\left( v_{x}\tau+\Delta_{x} \right)^{2}}{\omega^{2}\left( 1+\frac{\tau}{\tau_{D}} \right)} \right)exp\left( -\frac{\left( v_{y}\tau+\Delta_{y} \right)^{2}}{\omega^{2}\left( 1+\frac{\tau}{\tau_{D}} \right)} \right)exp\left( -\frac{\left( v_{z}\tau\right)^{2}}{{\alpha^{2}\omega}^{2}\left( 1+\frac{\tau}{{\alpha^{2}\tau}_{D}} \right)} \right)}{m\left( 1+\frac{\tau}{\tau_{D}} \right)\left( 1+\frac{\tau}{\alpha^{2}\tau_{D}} \right)^{\frac{1}{2}}\left( 1-exp\left( -\frac{\gamma}{2} \right)\sin\left( \frac{\gamma^{'}}{2} \right) \right)\left( 1-exp\left( -\frac{\gamma}{2} \right)\sin\left( \frac{\gamma^{'}}{2} - \varphi\right) \right)}\times\left\{ 1-exp\left( -\frac{\gamma\left( 1+\frac{2\tau}{\alpha^{2}\tau_{D}} \right)}{\left( 1+\frac{\tau}{\alpha^{2}\tau_{D}} \right)} \right)\sin\left( \frac{\sqrt{\gamma}\left( v_{z}\tau\right)}{\left( 1+\frac{\tau}{\alpha^{2}\tau_{D}} \right)}+\frac{\gamma'}{2} \right)+ exp\left( -\frac{\gamma\left( 1+\frac{2\tau}{\alpha^{2}\tau_{D}} \right)}{\left( 1+\frac{\tau}{\alpha^{2}\tau_{D}} \right)} \right)\sin\left( \frac{\sqrt{\gamma}\left( v_{z}\tau\right)}{\left( 1+\frac{\tau}{\alpha^{2}\tau_{D}} \right)}-\frac{\gamma'}{2}+\varphi\right)-\frac{1}{2}exp\left( -\gamma\right)\cos\left( \gamma'-\varphi\right) +\frac{1}{2}exp\left( -\frac{\gamma}{\left( 1+\frac{\tau}{\alpha^{2}\tau_{D}} \right)} \right)\cos\left( \frac{2\sqrt{\gamma}\left( v_{z}\tau\right)}{\left( 1+\frac{\tau}{\alpha^{2}\tau_{D}} \right)}+\varphi\right) \right\}$

**S5 text: Calculation of fluorescence signal**

For experimental correlation function the fluorescence signal is calculated by taking the integrated signal from the $m\times m$ pixel mask from which the background is subtracted from the entire $N\times N$ pixel that was imaged.

$$F=\sum_{i=1}^{m^{2}} F_{i}-m^{2}\left( \frac{\sum_{j=1}^{N^{2}} F_{j}}{N^{2}} \right)$$

where $F_{i}$ is the fluorescence signal from the pixels in the region of interest and $F_{j}$ is the fluorescence signal from the pixels in the entire image.
